# Anæsthetics in Labor

**Published:** 1883-09-20

**Authors:** E. Herriott

**Affiliations:** Jacksonville, Ill.


### ANÆSTHETICS IN LABOR.

BY E. HERRIOTT, M.D., JACKSONNILLE, ILL.

We expressed our views on this subject in a report to the Illinois State Medical Society in 1880, which embraced our experience of fifteen years with and without its use. Since then we have had no occasion to make any observation without, except where the patient or friends absolutely opposed, and this has been exceedingly limited. By their use the fears of the accoucheur, as to lacerations, exhaustion, and a train of troubles following, are to a great extent allayed by thoroughly relaxing the circular fibres of the os uteri, and all the soft parts, without arresting contractions of the body of the organ upon its contents; by allaying reflex irritation, allowing the organ to exert only its natural powers untrammeled, seldom calling into requisition the aid of the forceps; yet, when they are required, every preparation for their use has been completed, and no dread fears of them excite the patient. The annoying, cutting and exhausting pains before and during the relaxation of the os are abated; the dreaded agonies that are to follow are shorn of their terrors; finally, strength and tranquility reign supreme, instead of that crushed, lacerated and exhausted feeling from having had the arms almost torn from their sockets in the desire to aid the expulsive efforts. Hearing that welcome sound, the cry of her child, scarcely can she realize that the dreaded torture is over, for the time recently passed has been like a fairy dream to her. But enough on this branch, as it is found fully detailed in the Transactions of 1880, which contains practical facts.

As to the expulsion of the placenta, there is no period of labor where good judgment and skilled management are more important. Playfair says the cardinal point to bear in mind is, that it should be expelled by a *vis a tergo*, and not drawn out by a *vis a fronte.* Consequently, the estimate recently made on the average strength of the umbilical cord by Dr. Neville, of Ireland, is not of much advantage in this respect. In one hundred and twenty-five cords his experiments show, in those where the blood has been allowed to flow an average strength of twelve and five-twelfth pounds; where it was not allowed to flow, eleven pounds. After the expulsion of the child, the attendant seats himself by the side of the patient, places the hand over the abdomen, and gently grasps and kneads the organ until normal circnlation is established; then, grasping the organ more firmly about its center, squeezes continuously and firmly in a manner to expel the contents, keeping the fundus above the little finger and heel of the hand that the organs may be retained well up in position, while he makes very gentle traction on the cord with the other hand. After the expulsion, it should then be held and kneaded until it is found well contracted. The placenta expelled with caution in this manner is a material safeguard against hemorrhage. Nothing particularly new has been offered, within the past year, in the treatment of the latter. Lowering the head and shoulders, ice, alum, styptics with hot and cold water inside the womb, cold and kneading externally, ergot, iron, lead, etc., internally, electricity when convenient, grasping the os with the hand and stimulating it to contraction, digital pressure over the abdominal aorta, etc, are all well-known remedies to the profession.

One respondent much prefers the comp. liq. iodine, used on cotton lint and applied with the dressing forceps, to the preparations of persulphate of iron.

Very cold water poured in a small continuous stream over the abdomen readily produces capillary contraction; is a remedy that is always at hand, and that will produce, perhaps, as satisfactory results as any other single remedy, though one that seems to have been very much overlooked.—*Clinical Brief.*

